# Overexpression of miR -155 Promotes Proliferation and Invasion of Human Laryngeal Squamous Cell Carcinoma via Targeting SOCS1 and STAT3

**DOI:** 10.1371/journal.pone.0056395

**Published:** 2013-02-20

**Authors:** Xu-dong Zhao, Wei Zhang, Hong-jun Liang, Wen-yue Ji

**Affiliations:** 1 Department of Otorhinolaryngology, Shengjing Hospital, China Medical University, Shenyang, China; 2 Department of endocrinology Shengjing Hospital, China Medical University, Shenyang, China; 3 Educational Administration office, China Medical University, Shenyang, China; National Cancer Institute, National Institutes of Health, United States of America

## Abstract

MicroRNA155 plays an important role in many solid malignancies. Expression and function of miR-155 in laryngeal carcinoma have not been fully understood. This study aims to investigate the expression and function of miR-155 in laryngeal squamous cell carcinoma (LSCC), the relationship between miR-155 and its downstream target suppressor of cytokine signaling 1 (SOCS1)-STAT3 pathway, and the related clinicopathological factors. Sixty-three samples of laryngeal squamous cell carcinoma and twenty-one samples of control mucosa obtained from total laryngectomy cases were analyzed using Western blot analysis and real-time PCR. Hep-2 cells were cultured and transfected with miR-155 mimic and ASO. Cell proliferation, migration and invasion assays were used to determine the role of miR-155 in regulation of LSCC growth, migration, and invasion, respectively. The expression levels of miR-155 in LSCC were significantly higher than those in the control mucosa tissues. Downregulation of SOCS1 expression and elevated expression of STAT3 were also observed in LSCC. The relevance of the three factors were statistically significant. Moreover, knockdown of miR-155 elevated SOCS1expression level, suppressed STAT3 expression, and inhibited hep-2 cells growth, migration and invasion. Whereas overexpression of miR-155 inhibited SOCS1expression, elevated STAT3 expression, and promoted hep-2 cells growth, migration and invasion. Furthermore, the miR-155 levels in T_3_ T_4_ stages, and poor/moderate cell differentiation were significantly higher than those in T_2_ stage and higher degree of cell differentiation. The STAT3 protein in poor/moderate cell differentiation was significantly higher than those in higher degree of cell differentiation. We firstly demonstrated the aberrant expression and function of miR-155 and itsdownstream targets in LSCC. The current findings suggest that miR-155 play promotingrole during the development of LSCC, and miR-155 may be a useful marker for the prognosis and assessment of therapeutic effects.

## Introduction

Laryngeal cancer is the eleventh most common cancer in the worldwide. Laryngeal squamous cell carcinomas (LSCC) represent approximately 85–90% of all the malignant tumors of the larynx [1]. Although early-stage laryngeal cancer is often cured by surgery or radiotherapy, for the majority of patients with the advanced disease, the outcome has not improved in the last three decades. Thus the investigation of pathogenesis of LSCC is imperative in order to identify potential targets for therapeutic and prognosis purpose.

MicroRNAs (miRNAs, miRs) are endogenous, single-stranded, non-coding RNAs ranging from 18–24 nucleotides in length. They are highly conserved and ubiquitously expressed in all species [2]. microRNAs act as regulators of gene expression during development and differentiation at the transcriptional, posttranscriptional, and/or translational levels [3]. Recently, more and more evidence showed that aberrant expression of microRNAs had relationship with development of several human malignancies [4].

MicroRNA 155 (miR-155) is located on chromosome 21 and is transcribed from the B-cell integration cluster [5]. MiR-155 played an important role in many solid malignancies, including hepatocellular carcinoma, pancreatic cancer, lung cancer, breast cancer, colon cancer and nasopharyngeal carcinoma [6]–[11]. Recently, a new investigation reported that miR-155 functions as an oncomicroRNA in breast cancer,which decrease the expression of suppressor of cytokine signaling 1 (SOCS1) in breast cancer cell lines. And the research also showed overexpression of miR-155 in breast cancer cells leads to constitutive activation of signal transducer and activator of transcription 3 (STAT3) by inhibiting SOCS1 expression [12]. Activation of STAT3 may contribute to development of LSCC [13]. Another study demonstrated that the expression of SOCS1 and LR4-NFκB pathway molecules had a strong association with the aggressiveness of laryngeal carcinoma [14]. These observations strongly suggest that aberrant miR-155 expression, which targets SOCS1, may affect STAT3 pathway and play promoting effect during the development of LSCC. However, the significance of miR-155 expression in the prognosis of LSCC is still elusive. In this investigation, we demonstrated that hypothesis and identified the promoting effect of miR-155 during the development of LSCC. This investigation indicated a new pathway for the laryngeal tumorigenesis and prediction of the outcome.

## Materials and Methods

### Tissue Specimens

The study group consisted of 63 patients with LSCC diagnosed and treated between May 2008 and August 2010 at the Shengjing Hospital of China Medical University. The information about clinical samples and diagnoses were summarized in table S1. After having obtained informed consent from the patients and approval from the ethics committee, we collected the tumor samples. All the patients were diagnosed pathologically as having LSCC before the operation. All the patients were absence of distant metastases. No chemotherapy and radiation were applied to the patients before the operation. Among them, 21 patients underwent total laryngectomy, and 42 underwent partial laryngectomy. Control mucosa samples were obtained from 21 patients who received a total laryngectomy, over 2.0 cm away from the margin of the tumor. All the control mucosa samples used in this study were histologically normal. All the samples were immediately snap-frozen in liquid nitrogen and then stored in −80°C freezer. Relevant data (age, gender, primary tumor site and T stage of tumor, as well as differentiation grade of tumor) of the patients were extracted from the patients’ files. Staging of the tumors was carried out according to the UICC (International Union against Cancer) 2002 TNM classification. Differentiation status of tumor were determined pathologically by tumor cell morphology. Tumor cell morphology conforms to squamous cell structure and numbers of keratosic pearls exist were defined as high differentiation. Tumor cell morphology conforms to squamous cell structure but no keratosic pearls exist were defined as moderate differentiation. Low differentiation meant cell morphology was irregular and no squamous cell structure existed.

### Cell Culture and Transfection

Hep-2 cells were cultured in DMEM (GIBCO, USA) containing 10% fetal bovine serum at 37°C in a humidified atmosphere composed of 95% air and 5% CO2. The cells were grown overnight and then transfected with either miR-155 mimic, antisense 2′O-methyl oligonucleotides (ASO) against miR-155 (Genepharma, China),or their negative control miRNAs (Genepharma, China) by using Lipofectamine 2000 (Invitrogen, USA) according to the manufacturer’s protocol. The sequences of these oligonucleotides were as follows: miR-155 mimic:

Sense: 5′-UUA AUG CUA AUC GUG AUA GGG GU-3′,

Antisense: 5′-CCC UAU CAC GAU UAG CAU UAA UU-3′;

miR-155 mimic negative control:

sense: 5′-UUC UCC GAA CGU GUC ACG UTT-3′.

antisense: 5′-ACG UGA CAC GUU CGG AGA ATT-3′.

ASO: 5′-ACC CCU AUC ACG AUU AGC AUU AA-3′.

ASO negative control: 5′-CAG UAC UUU UGU GUA GUA CAA-3′.

Empty liposomes were also used as controls (liposome control) to transfect with Hep-2 cells.

### Quantitative Real-time PCR (qRT-PCR)

The quantification of miR-155 expression was carried out by TaqMan MicroRNA Assays kit (Ambion, USA) according to manufacturer’s protocol. Briefly, small RNAs were isolated from LSCC and control mucosa using the mirVanaTM miRNA isolation kit (Ambion, USA), and PCR was performed by the mirVanaTM miRNA detection kit (Ambion, USA) with miR-155 specific primer (Ambion, USA). U6 RNA was used as internal control in the qRT-PCR assay. Real-time PCR was performed in a LightCycler 3 instrument (Roche).All experiments were conducted in triplicates and repeated three times. The relative expression level was determined as 2^−ΔΔCt^. Data were presented as the expression level relative to the calibrator (mean value of control samples), with the standard error of the mean of triplicate measures for each test sample.

### Western Blotting

100 mg of tissue was added to 500 µL of general protein lysate (Galen, USA) for protein extraction. Twenty micrograms of protein diluted in NuPAGE sample buffer were denatured at 95°C for 5 min. The samples were loaded on a 8% SDSPAGE gel. Following electrophoresis, the proteins were blotted onto polyvinylidene difluoride membranes at 100 V for 70 min (Millipore, USA). Then the membranes were blocked in 5% skim milk for 1 h at room temperature. The membranes were incubated with either mouse anti-human STAT3 antibody (diluted 1∶400; BD Biosciences, USA) or rabbit anti-human SOCS1 antibody (diluted 1∶400; Abcam, USA) or rabbit anti-human β-actin antibody (diluted 1∶800; Santa Cruz Biotechnology, USA) at 4°C overnight. After extensive washing, they were incubated with secondary horseradish peroxidase-conjugated anti-mouse or anti-rabbit antibodies (diluted 1∶2,000; Amersham Biosciences, UK). After several washing steps, nitrocellulose membranes were incubated with the Western blotting detection system ECL (Amersham Biosciences).

### Cell Proliferation Assay

36 h after transient transfection, Hep-2 were harvested and sub-cultured in 96-well plates for up to 96 h. After that, cell proliferation was assessed using the Cell-Titer 96 AQueous MTS assay ((Promega, USA) according to the manufacturer’s protocol. The MTS reagent (20 µl) was added to each well and incubated at 37°C for 4 h. Then, the absorbance at 492 nm was measured by using a microtiter plate reader (Bio-Rad, CA).The experiments were in triplicate and repeated thrice. The data were summarized as mean ± SD.

### Cell Migration and Invasion Assay

Tumor cell migration and invasion were carried out using a Transwell insert (8 µm, Corning, USA). Hep-2 cells were transfected with miR-155 mimic, ASO or their NC. After 48 h incubation, the cells were starved in a medium without fetal bovine serum overnight, and then 1×10^5^ cells resuspended in 0.1 ml serum-free medium were added to the upper chamber and DMEM containing 10% fetal bovine serum was added to the lower chamber as a chemoattractant. For the invasion assay, the inserts were precoated with extracellular Matrigel (BD Biosciences, USA). The cells in the upper chamber were cultured for 24 h on Hep-2 cells to measure the effect of miR-155 mimic and ASO transfection on Hep-2 invasion and migration potential. Invaded or migrated cells were fixed and stained with 0.1% crystal violet. Five low-magnification areas (x100) were randomly selected and counted for the cell numbers. All experiments were performed in triplicate.

### Statistical Analysis

All statistical analyses were carried out using SPSS version 17.0 (Statistical Package for the Social Sciences). The experiments were conducted in triplicates. The levels of SOCS1 and STAT3 protein were expressed by (SOCS1 and STAT3 protein gray scale value/β-actin gray scale value) × 100% Measurement data were expressed as mean ±SD. All the parameters recorded in tumor material passed tests for being normally distributed (P-P graph test) and therefore the expression difference of miR-155, SOCS1 and STAT3 were analyzed by the t test. And Pearson test were used for analyzing the relationship between miR-155, SOCS1 and STAT3 expression. The correlations between miR-155 expression and clinicopathological parameters were also statistically analyzed by the t test. P-values<0.05 was considered significant.

## Results

### MiR-155 was Detected at Higher Level in LSCC

**Figure 1:**
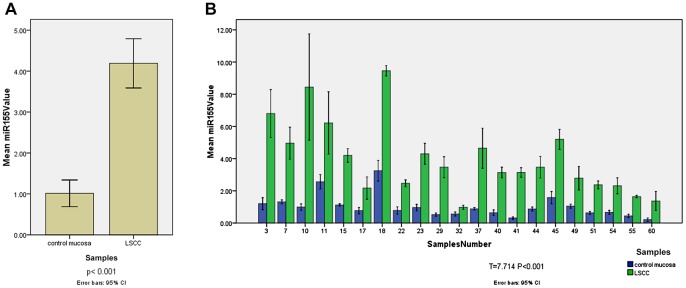
**Figure 1A:** The miR-155 expression difference between LSCC and control mucosa. The expression levels of miR-155 in sixty-three samples of laryngeal squamous cell carcinoma and twenty-one samples of control mucosa were analyzed by real-time PCR.U6 RNA was used as internal control. The relative expression level was determined as 2^−ΔΔCt^. The relative values of miR-155 in LSCC were 4.19±2.39; the values of miR-155 in the control mucosa were 1.01±0.72. The expression levels of miR-155 in LSCC were 4.1-fold higher than those in the control mucosa tissues (p<0.001). **Figure 1B:** **The miR-155 expression difference between pairwised tumor and control mucosa specimens.** MiR-155 expression level was compared between 21 pairwised tumor and control mucosa specimens obtained from 21 patients who received total laryngectomy. The miR-155 expression difference between 21 pairwised tumor and control mucosa specimens was statistically significant (T = 7.714, P<0.001).

Quantitative real-time PCR was performed to evaluate miR-155 expression in 63 tumor and 21 control mucosa specimens (Fig.1A). The relative values of miR-155 in LSCC were 4.19±2.39; the values of miR-155 in the control mucosa were 1.01±0.72. The expression levels of miR-155 in LSCC were 4.1-fold higher than those in the control mucosa tissues; and the difference was statistically significant (p<0.001). MiR-155 expression were also compared between 21 pairwised tumor and control mucosa specimens obtained from 21 patients who received total laryngectomy (Fig.1B). The miR-155 expression difference between 21 pairwised tumor and control mucosa specimens was statistically significant (T = 7.714, P<0.001).

### Downregulation of SOCS1 Expression in LSCC

**Figure 2:**
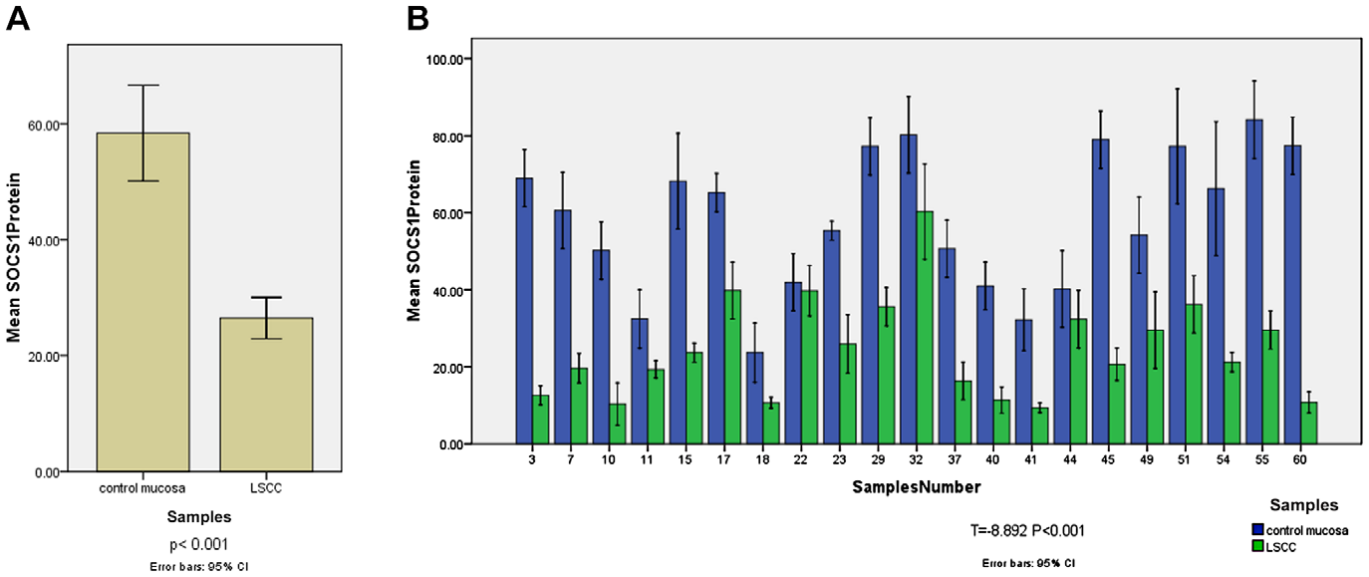
**Figure 2A:** The SOCS1 expression difference between LSCC and control mucosa. The protein levels of SOCS1 in sixty-three samples of laryngeal squamous cell carcinoma and twenty-one samples of control mucosa were analyzed by western blot. The values of SOCS1 protein were 26.49±14.21in tumor specimens, and 58.40±18.13 in control mucosa specimens. The protein levels of SOCS1 in control mucosa tissues were 2.2-fold higher than those in tumor tissues (p<0.001). **Figure 2B:** **The SOCS1 expression difference between pairwised tumor and control mucosa specimens.** SOCS1 protein expression were compared between 21 pairwised tumor and control mucosa specimens obtained from 21 patients who received total laryngectomy. The SOCS1 protein expression difference between 21 pairwised tumor and control mucosa specimens was statistically significant (T = −8.892, P<0.001).

In order to investigate the SOCS1 protein expression level in LSCC, we used western blot to detect SOCS1 in 63 tumor and 21 control mucosa specimens (Fig.2A). The values of SOCS1 protein were 26.49±14.21 in tumor specimens, and 58.40±18.13 in control mucosa specimens. The protein levels of SOCS1 in control mucosa tissues were 2.2-fold higher than those in tumor tissues; the differences were statistically significant (p<0.001). SOCS1 protein expression was also compared between 21 pairwised tumor and control mucosa specimens obtained from 21 patients who received total laryngectomy (Fig.2B). The SOCS1 protein expression difference between 21 pairwised tumor and control mucosa specimens was statistically significant (T = -8.892, P<0.001).

### STAT3 Protein Levels were Elevated in LSCC Specimens

**Figure 3:**
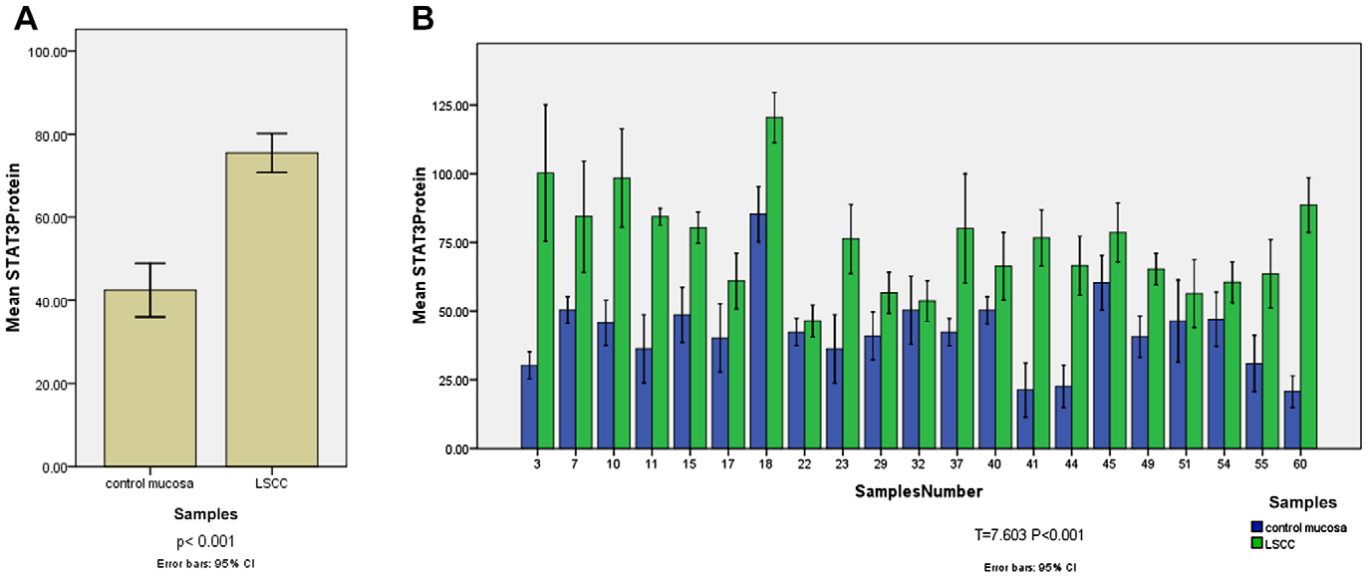
**Figure 3A:** The STAT3 expression difference between LSCC and control mucosa. The protein levels of STAT3 in sixty-three samples of laryngeal squamous cell carcinoma and twenty-one samples of control mucosa were analyzed by western blot. The values of STAT3 protein were 75.47±18.71 in tumor specimens, and 42.46±14.15 in control mucosa specimens. The protein levels of STAT3 in tumor tissues were 1.8-fold higher than those in control mucosa tissues (p<0.001). **Figure 3B:** **The STAT3 expression difference between pairwised tumor and control mucosa specimens.** STAT3 protein expression were compared between 21 pairwised tumor and control mucosa specimens obtained from 21 patients who received total laryngectomy. The STAT3 protein expression difference between 21 pairwised tumor and control mucosa specimens was statistically significant (T = 7.603, P<0.001).

Western blot was used to detect STAT3 protein expression in 63 tumor and 21 control mucosa specimens (Fig.3). The values of STAT3 protein were 75.47±18.71 in tumor specimens, and 42.46±14.15 in control mucosa specimens. The protein levels of STAT3 in tumor tissues were 1.8-fold higher than those in control mucosa tissues; the differences were statistically significant (p<0.001). STAT3 protein expression was also compared between 21 pairwised tumor and control mucosa specimens obtained from 21 patients who received total laryngectomy (Fig.3B). The STAT3 protein expression difference between 21 pairwised tumor and control mucosa specimens was statistically significant (T = 7.603, P<0.001).

### Association between miR-155, SOCS1 and STAT3

**Figure 4:**
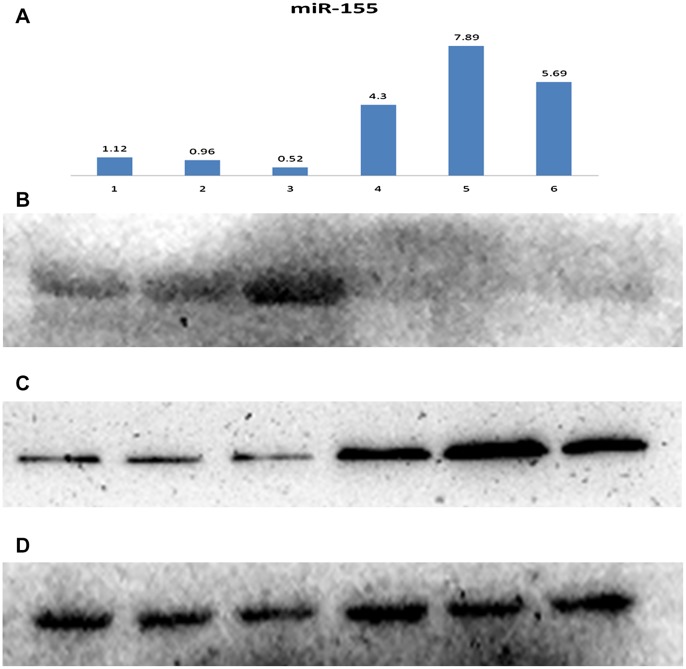
The relationship between miR-155, SOCS1 and STAT3. The correlation between miR-155, SOCS1 and STAT3 in 63 tumor and 21 control mucosa specimens were analyzed. As we expected, negative correlation were found between miR-155, SOCS1 and STAT3. The correlation between miR-155, SOCS1 and STAT3 in tumor and control mucosa specimens obtained from representative NO.7, 18 and 32 patients (stage II,III and IV,respectively ) who received total laryngectomy were shown in figure 4. MiR-155 had a significant negative correlation with SOCS1 protein, and also a significant positive correlation with STAT3 protein.

**Figure 5:**
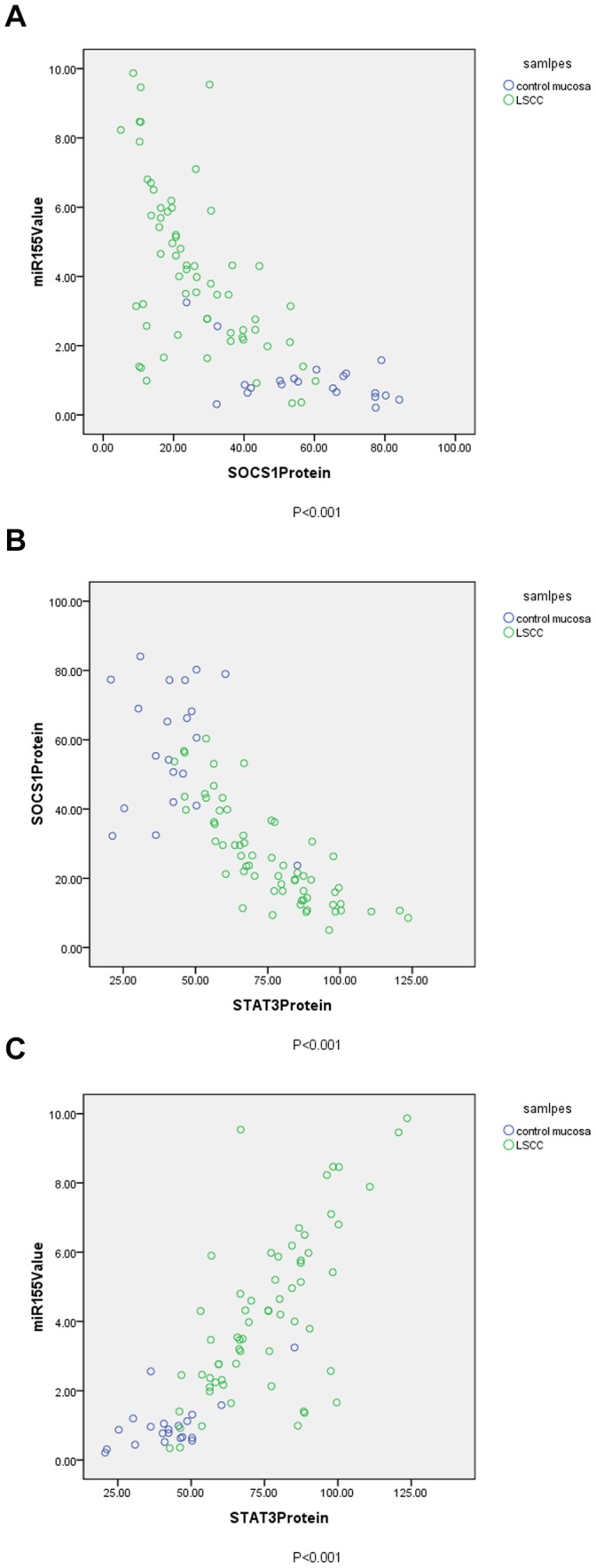
**Figure 5A:** The correlation between miR-155 and SOCS1. The correlation between miR-155 and SOCS1 in 63 tumor and 21 control mucosa specimens were analyzed. MiR-155 had a significant negative correlation with SOCS1 protein. Pearson correlation coefficient was −0.691 (p<0.001). **Figure 5B:** **The correlation between SOCS1 and STAT3.** The correlation between SOCS1 and STAT3 in 63 tumor and 21 control mucosa specimens were also analyzed. Significant negative correlation was found between SOCS1 and STAT3. Pearson correlation coefficient was −0.787 (p<0.001). **Figure 5C:** **The correlation between miR-155 and STAT3.** The correlation between miR-155 and STAT3 in 63 tumor and 21 control mucosa specimens were analyzed. MiR-155 had a significant positive correlation with STAT3 protein. Pearson correlation coefficient was 0.773 (p<0.001).

In our investigation we evaluated the correlation between miR-155, SOCS1 and STAT3 in 63 tumor and 21 control mucosa specimens. As we expected, negative correlation were found between miR-155, SOCS1 and STAT3 (Fig.4). MiR-155 had a significant negative correlation with SOCS1 protein. Pearson correlation coefficient was -0.691 (p<0.001) (Fig.5A). SOCS1 had a significant negative correlation with STAT3 protein. Pearson correlation coefficient was −0.787 (p<0.001) (Fig.5B). MiR-155 had a significant positive correlation with STAT3 protein. Pearson correlation coefficient was 0.773 (p<0.001) (Fig.5C).

### Effect of miR-155 on Hep-2 Cell Growth

**Figure 6:**
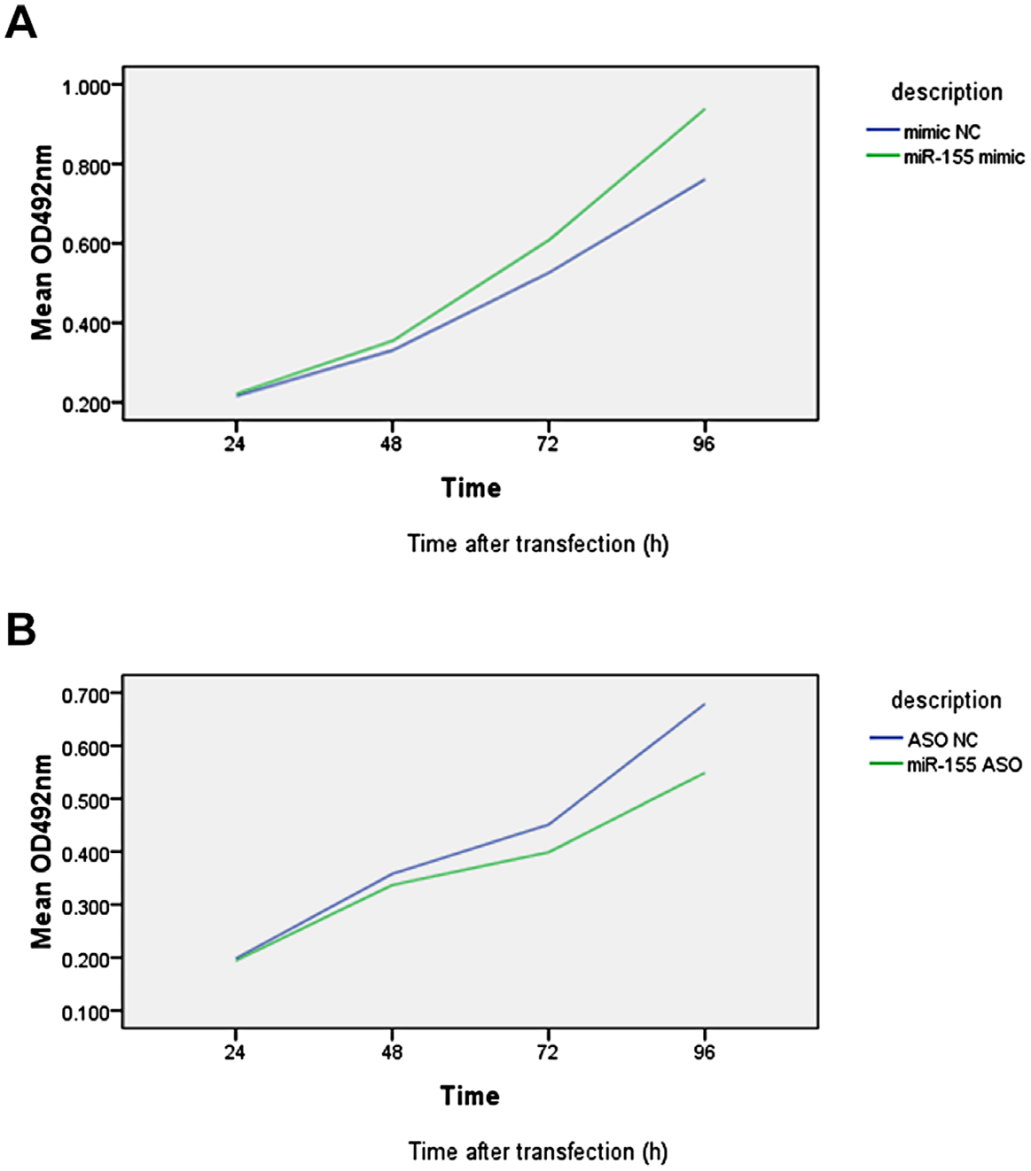
**Figure 6A:** **Cell proliferation assay in Hep-2 cells transfected with miR-155 mimic or NC.** Hep-2 cells were grown and transiently transfected with miR-155 mimic or NC, and cell proliferation was then assessed afterwards. The experiments were performed in triplicate and repeated thrice. Compared with NC group, miR-155 mimic-transfected cells showed a time-dependent increase of cell proliferation, i.e., rates of growth increase were 2.9%, 7.2%, 15.5% and 23.2% for 24 h, 48 h, 72 h, and 96 h after gene transfection, respectively (P<0.05). **Figure 6B:** **Cell proliferation assay in Hep-2 cells transfected with miR-155 ASO or NC.** Cell proliferation assay. Hep-2 cells were grown and transiently transfected with miR-155 ASO or NC, and cell proliferation was then assessed afterwards. The experiments were performed in triplicate and repeated thrice. Compared with NC group, cells transfected with miR-155 ASO showed a time-dependent reduction of cell proliferation, i.e., rates of growth inhibition were 1.7%, 5.9%, 11.5% and 19.2% for 24 h, 48 h, 72 h, and 96 h after gene transfection, respectively (P<0.05).

To evaluate the effect on cell proliferation, Hep-2 cells were transiently transfected with miR-155 mimic, miR-155 ASO or their NC. Compared with NC group, miR-155 mimic-transfected cells showed a time-dependent increase of cell proliferation, i.e., rates of growth increase were 2.9%, 7.2%, 15.5% and 23.2% for 24 h, 48 h, 72 h, and 96 h after gene transfection, respectively (P<0.05, Fig.6A). In contrast, cells transfected with miR-155 ASO showed a time-dependent reduction of cell proliferation, i.e., rates of growth inhibition were 1.7%, 5.9%, 11.5% and 19.2% for 24 h, 48 h, 72 h, and 96 h after gene transfection, respectively (P<0.05, Fig.6B).

### Effects of miR-155 on Cancer Cell Migration and Invasion

**Figure 7:**
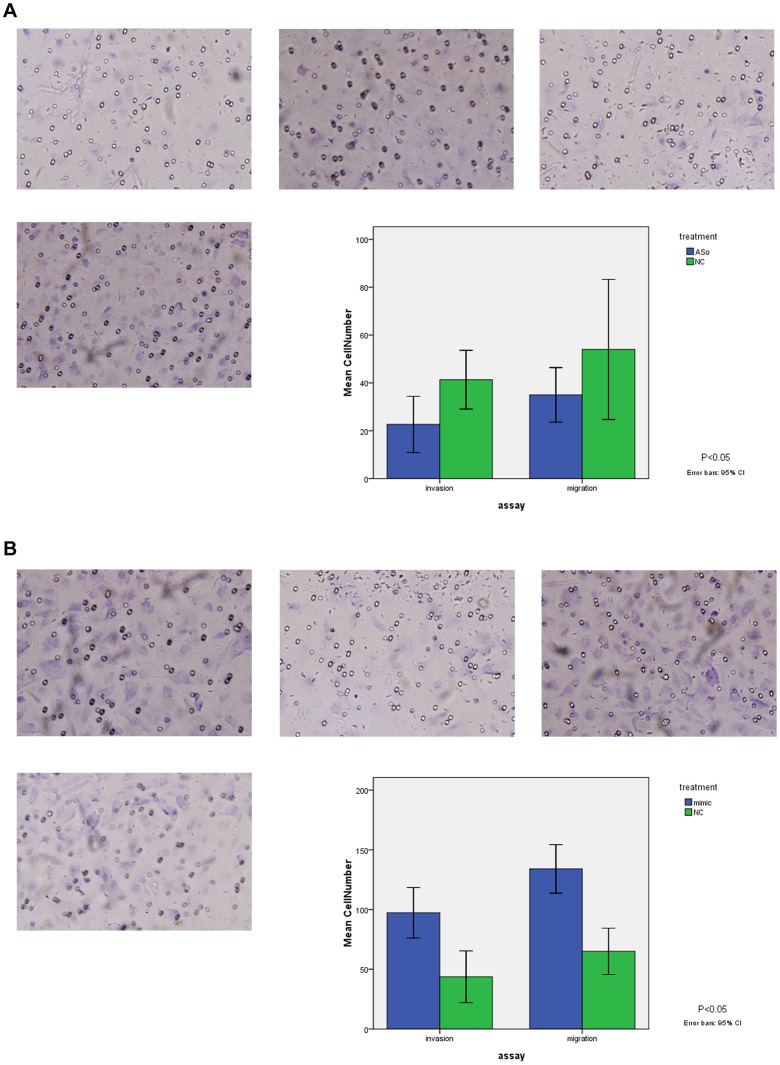
**Figure 7A:** Transwell migration and Matrigel invasion assays. Hep-2 cells were grown and transiently transfected with miR-155 ASO and NC subjected to migration and invasion assays. Representative photographs (upper) and quantification (lower) are shown. Magnification: × 200. **Figure 7B:** **Transwell migration and Matrigel invasion assays. Hep-2 cells were grown and transiently transfected with miR-155 mimic and NC subjected to migration and invasion assays.** Representative photographs (upper) and quantification (lower) are shown. Magnification: × 200.

The potential impact of miR-155 on cell migration and invasion were assessed using transwell migration and invasion assays. After Hep-2 cells was selected for overexpression and knockdown of miR-155 using transient transfection, transwell migration and Matrigel invasion assays data showed that miR-155 knockdown resulted in reduction of hep-2 cells migration (P<0.05) and invasion rate (P<0.05) compared with NC group (Fig.7A). In contrast, miR-155 overexpression resulted in increase of hep-2 cells migration (P<0.05) and invasion rate (P<0.05) compared with NC group (Fig.7B).

### Knockdown and Overexpression of miR-155 Change Expression Levels of SOCS1 and STAT3 Protein

**Figure 8:**
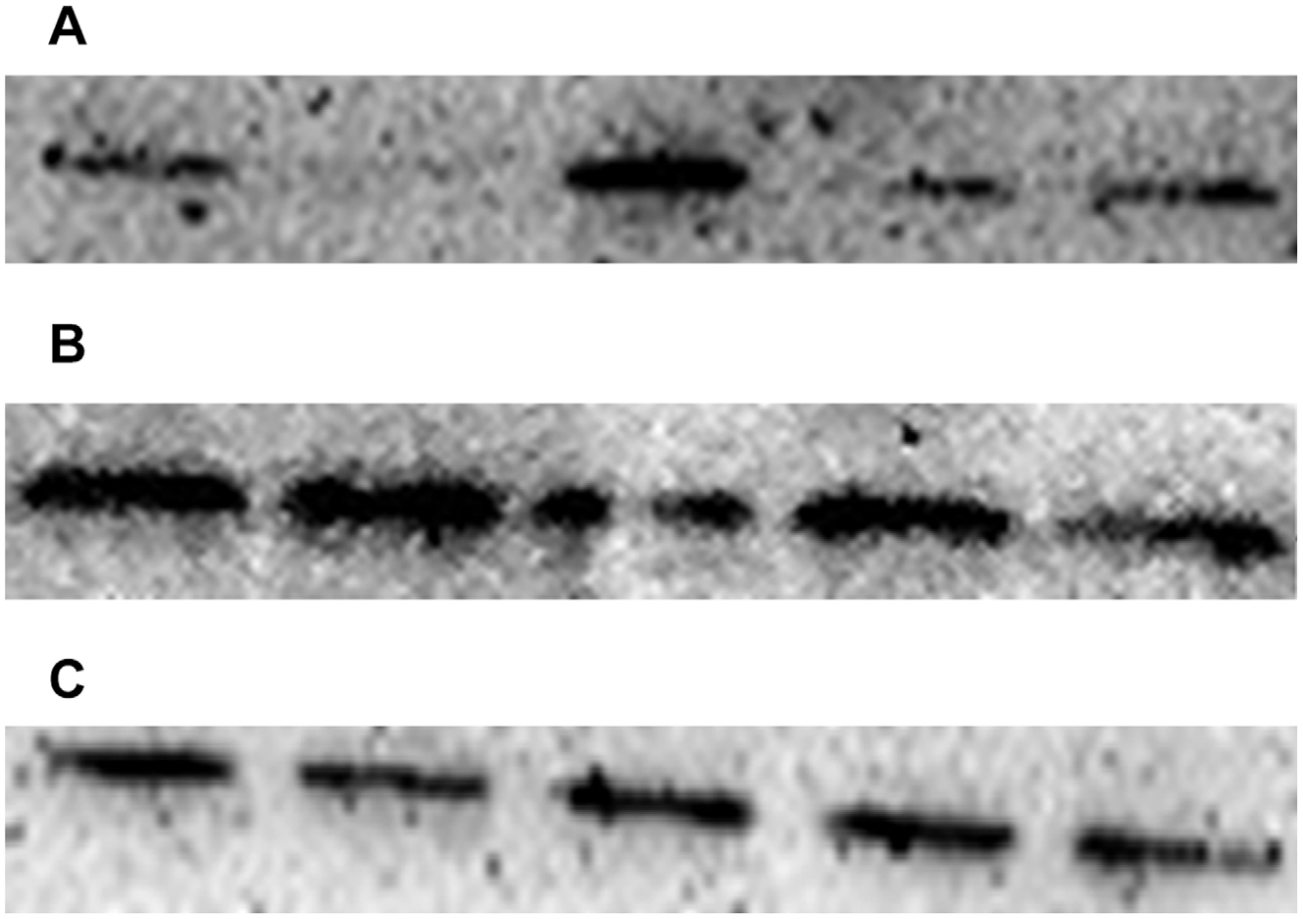
SOCS1 and STAT3 expression in Hep-2 cells transfected with miR-155 mimic, miR-155 ASO or their negative controls. SOCS1 protein expression was inhibited 57% by transfection with miR-155 mimic (p<0.05). In contrast, after knockdown of miR-155 by transfection of miR-155 ASO, the SOCS1 level increased to 2.26 fold (p<0.05). STAT3 protein level increase to 1.41 fold after transfection with miR-155 mimic(p<0.05). In contrast, after knockdown of miR-155 by transfection of miR-155 ASO, STAT3 protein expression was inhibited 38% (p<0.05).

Hep-2 cells were grown and transfected with miR-155 mimic, miR-155 ASO or their negative controls. The expression change of SOCS1 and STAT3 protein of hep-2 cells caused by knockdown and overexpression of miR-155 were evaluated by western blot (Fig.8). All experiments were conducted in triplicates and repeated three times. The data were summarized as mean ± SD.

The SOCS1 expression level of hep-2 cells was 30.84±15.66, whereas after transfection of miR-155 mimic, the SOCS1 protein level decrease to 13.26±8.72. SOCS1 protein expression was inhibited 57%. The SOCS1 protein expression difference was statistically significant (p<0.05). The SOCS1 protein level of hep-2 cells transfected with mimic NC was 25.29±11.83. In contrast, after knockdown of miR-155 by transfection of miR-155 ASO, the SOCS1 level increased to 69.73±16.39. SOCS1 protein expression increased to 2.26 fold. The SOCS1 protein expression difference was statistically significant (p<0.05). The SOCS1 protein level of hep-2 cells transfected with ASO NC was 39.61±12.83.

The STAT3 expression level of hep-2 cells was 83.14±12.63, whereas after transfection of miR-155 mimic, the STAT3 level increased to 117.13±34.96. STAT3 protein level increase to 1.41 fold. The STAT3 protein expression difference was statistically significant (p<0.05). The STAT3 protein level of hep-2 cells transfected with miR-155 mimic NC was 85.25±18.94. In contrast, after knockdown of miR-155 by transfection of miR-155 ASO, the STAT3 level decreased to 51.61±18.22. STAT3 protein expression was inhibited 38%. The STAT3 protein expression difference was statistically significant (p<0.05). The STAT3 protein level of hep-2 cells transfected with ASO NC was 73.90±16.10.

### Relationship between Different Clinicopathological Parameters and miR-155, SOCS1 and STAT3

The miR-155 values in different sex, age, cell differentiation, primary tumor site and T stage were analyzed by SPSS version 17.0 to determine if the miR-155 expression was correlated with these parameters. The results are shown in table 1. The miR-155 levels in different sex, age, and tumor site had no statistically significant difference. The miR-155 levels in T_3_ T_4_ stages, and poor/moderate cell differentiation were significantly higher than those in T_2_ stage and higher degree of cell differentiation. The SOCS1 and STAT3 protein expression level in different T stage and cell differentiation were also analyzed. The results are shown in table 2. The expression of SOCS1 protein in different T stages and cell differentiation had no statistically significant difference. The STAT3 protein in poor/moderate cell differentiation were significantly higher than those in higher degree of cell differentiation. The expression of STAT3 protein in different T stages had no statistically significant difference.

**Table 1:** Correlation between miR-155 and clinicopathological parameters.

Factors	Patients Number	miR-155	p value	
Total	63			
Gender			0.880	
Male	57(90.5)	4.18±2.43		
Female	6(9.5)	4.33±2.08		
Age(Y)			0.885	
≧60	35(55.6)	4.15±2.43		
<60	28(44.4)	4.24±2.38		
Primary Site			0.795	
Glottic	38(60.3)	4.25±2.25		
Supraglottic	25(39.7)	4.09±2.62		
T Stage			0.014	
T_2_	29(46.0)	3.40±2.10		
T_3_ T_4_	34(54.0)	4.86±2.44		
Differentiation			0.021	
High	37(58.7)	3.61±1.70		
Moderate and low	26(41.3)	5.01±2.96		

Values in parentheses represent percentage.

**Table 2:** Correlation between SOCS1, STAT3 and clinicopathological parameters.

Factors	SOCS1	p value	STAT3	p value	
T Stage		0.777		0.145	
T_2_	25.93±12.69		71.73±15.45		
T_3_ T_4_	26.96±15.57		78.65±20.79		
Differentiation		0.144		0.002	
High	28.69±12.63		69.60±13.23		
Moderate andlow	23.36±15.93		83.82±22.18		

## Discussion

The development of LSCC is clearly a multistep process involving multiple factors. Up to now, most investigations to this malignancy have focused on molecular changes occurring in cell signals and on genomic changes. In the current study, we focused on a different aspect of LSCC pathogenesis in the role of miR-155 in the process. It is reported firstly that miR-155 had a close relation with human B cell lymphomas [15]. MiR-155 played an important role in many solid malignancies [6]–[11]. Recently, a new study found that miR-155 targeting suppressor of cytokine signaling 1 (SOCS1) who inhibit STAT3 functions as an oncomicroRNA in a breast cancer cell line [12]. Moreover, SOCS1 having the capability of suppressing cytokine signal transduction was reported firstly by Starr et al. [16]. Many investigations indicated that SOCS1 played an important role in many malignancies, including colon cancer, liver cancer, lung cancer, pancreatic cancer and breast cancer[10], [17]–[20]. Furthermore, Starska et al demonstrated that SOCS1 have a strong association with the aggressiveness of laryngeal carcinoma [14]. STAT3 is an important member in STATs, which is activated mainly through tyrosine 705 phosphorylation by Janus protein-tyrosine kinases. Activated STAT3 forms homodimer or heterodimer, which translocates into nucleus and binds with target sequences to increase target gene transcription [21]. STAT3 had been reported to be an oncogene in a variety of human malignancies including LSCC [13], [22]–[24]. Moreover JAK2/STAT3 signaling were suppressed by SOCS1 in many malignancies [25]–[28]. Based on these investigations, we put forward the hypothesis that aberrant miR-155 expression, which targets SOCS1, may affect STAT3 pathway and play some role during the development of LSCC.

In our study we demonstrated the significantly elevated expression of miR-155 and STAT3, also the significantly decreased expression of SOCS1 in LSCC. The aberrant expression of miR-155 in LSCC was found by the first time. In addition, we discovered that miR-155 had a significant negative correlation with SOCS1 protein, and SOCS1 protein had a significant negative correlation with STAT3 protein in LSCCs. Moreover, knockdown of miR-155 elevated SOCS1expression level, suppressed STAT3 expression, and inhibited hep-2 cells growth, migration and invasion. Whereas overexpression of miR-155 inhibited SOCS1expression, elevated STAT3 expression, and promoted hep-2 cells growth, migration and invasion. Furthermore, we observed the higher miR-155 levels in LSCC of advanced T stages (T3 and T4) compared with those of early T stage (T2), and a negative correlation between miR-155 level and tumor cell differentiation for the first time. Aslo, the STAT3 protein in poor/moderate cell differentiation was found significantly higher than those in higher degree of cell differentiation. These results indicated that aberrant miR-155 expression, which targets SOCS1, may affect STAT3 and promote the development of LSCC.

Our study did not include LSCC of T1 because all T1 LSCC had been treated by lasers. Collectively, our results have significance for understanding a new pathway of the development of LSCC and for prognosis assessment. Targeting miR-155 in LSCC may provide an effective therapeutic approach to treat laryngeal cancer.

### Conclusions

MiR-155 play promotingrole during the development of LSCC, and miR-155 may be a useful marker for the prognosis and assessment of therapeutic effects.

## Supporting Information

Table S1 **The information about clinical samples and diagnoses.** The clinical information of the samples were summarized in table S1.
(DOC)
