# Supplementary material for: Overexpression of miR -155 Promotes Proliferation and Invasion of Human Laryngeal Squamous Cell Carcinoma via Targeting SOCS1 and STAT3
Source: PLoS One. 2013 Feb 20;8(2):e56395. doi: 10.1371/journal.pone.0056395 (PMC3577898; doi:10.1371/journal.pone.0056395)
Supplement: Table S1 — The information about clinical samples and diagnoses. The clinical information of the samples were summarized in table S1. (DOC) [file pone.0056395.s001.doc]

Supplement Table 1. The information about clinical samples and diagnoses

| **Sample** | **gender** | **age** | **primary cite** | | | **diagnosis** | **differentiation** | |
| --- | --- | --- | --- | --- | --- | --- | --- | --- |
| **1** | male | 54 | supraglottic | | T3N0M0 III stage | | | moderate |
| **2** | male | 63 | glottic | | | T2N0M0 II stage | | high |
| **3** | male | 65 | supraglottic | | | T4N1M0 IV stage | | low |
| **4** | male | 52 | | glottic | | T2N0M0 II stage | | moderate |
| **5** | male | 49 | glottic | | | T3N0M0 III stage | | high |
| **6** | female | 68 | supraglottic | | | T2N1M0 III stage | | high |
| **7** | male | 58 | Supraglottic | | | T2N0M0 II stage | | high |
| **8** | male | 73 | supraglottic | | | T3N2M0 IV stage | | moderate |
| **9** | female | 53 | glottic | | | T3N0M0 III stage | | high |
| **10** | male | 67 | glottic | | | T4N0M0 IV stage | | moderate |
| **11** | male | 72 | | glottic | | T2N0M0 II stage | | high |
| **12** | male | 55 | | glottic | | T2N0M0 II stage | | high |
| **13** | male | 52 | glottic | | | T3N0M0 III stage | | low |
| **14** | female | 78 | supraglottic | | | T3N0M0 III stage | | high |
| **15** | male | 58 | supraglottic | | | T4N1M0 IV stage | | low |
| **16** | male | 69 | | glottic | | T2N0M0 II stage | | high |
| **17** | male | 77 | | glottic | | T2N0M0 II stage | | high |
| **18** | male | 48 | supraglottic | | | T3N0M0 III stage | | moderate |
| **19** | male | 56 | | glottic | | T2N0M0 II stage | | moderate |
| **20** | male | 73 | glottic | | | T3N0M0 III stage | | high |
| **21** | male | 56 | glottic | | | T2N0M0 II stage | | high |
| **22** | male | 64 | glottic | | | T3N0M0 III stage | | moderate |
| **23** | male | 68 | glottic | | | T3N1M0 III stage | | moderate |
| **24** | female | 76 | glottic | | | T3N0M0 III stage | | low |
| **25** | male | 50 | | glottic | | T3N0M0 III stage | | high |
| **26** | female | 54 | | glottic | | T2N0M0 II stage | | moderate |
| **27** | male | 67 | | glottic | | T2N0M0 II stage | | high |
| **28** | male | 59 | | glottic | | T3N0M0 III stage | | moderate |
| **29** | male | 74 | | glottic | | T3N0M0 III stage | | moderate |
| **30** | male | 66 | supraglottic | | | T2N0M0 II stage | | high |
| **31** | male | 72 | | glottic | | T3N0M0 III stage | | low |
| **32** | male | 55 | | glottic | | T4N0M0 IV stage | | moderate |
| **33** | male | 53 | | glottic | | T2N0M0 II stage | | high |
| **34** | male | 74 | supraglottic | | | T2N0M0 II stage | | high |
| **35** | male | 62 | supraglottic | | | T2N0M0 II stage | | high |
| **36** | male | 71 | | glottic | | T3N0M0 III stage | | moderate |
| **37** | male | 54 | supraglottic | | | T3N1M0 III stage | | moderate |
| **38** | male | 69 | supraglottic | | | T2N0M0 II stage | | high |
| **39** | male | 55 | | glottic | | T2N0M0 II stage | | moderate |
| **40** | male | 76 | | glottic | | T2N0M0 II stage | | high |
| **41** | male | 64 | supraglottic | | | T3N1M0 III stage | | low |
| **42** | male | 57 | | glottic | | T2N0M0 II stage | | high |
| **43** | male | 52 | supraglottic | | | T3N0M0 III stage | | high |
| **44** | male | 70 | supraglottic | | | T3N0M0 III stage | | moderate |
| **45** | male | 64 | supraglottic | | | T3N0M0 III stage | | high |
| **46** | male | 66 | | glottic | | T2N0M0 II stage | | moderate |
| **47** | male | 49 | | glottic | | T2N0M0 II stage | | high |
| **48** | male | 47 | glottic | | | T3N0M0 III stage | | high |
| **49** | male | 69 | supraglottic | | | T2N0M0 II stage | | high |
| **50** | female | 56 | supraglottic | | | T2N0M0 II stage | | high |
| **51** | male | 65 | | glottic | | T4N0M0 IV stage | | moderate |
| **52** | male | 76 | | glottic | | T3N0M0 III stage | | high |
| **53** | male | 70 | supraglottic | | | T2N0M0 II stage | | high |
| **54** | male | 55 | supraglottic | | | T3N1M0 III stage | | high |
| **55** | male | 58 | | glottic | | T3N1M0 III stage | | high |
| **56** | male | 52 | supraglottic | | | T2N0M0 II stage | | high |
| **57** | male | 69 | supraglottic | | | T2N0M0 II stage | | high |
| **58** | male | 62 | | glottic | | T2N0M0 II stage | | moderate |
| **59** | male | 56 | | glottic | | T3N0M0 III stage | | high |
| **60** | male | 51 | supraglottic | | | T3N1M0 III stage | | high |
| **61** | male | 73 | | glottic | | T2N0M0 II stage | | low |
| **62** | male | 69 | | glottic | | T3N0M0 III stage | | high |
| **63** | male | 64 | supraglottic | | | T3N0M0 III stage | | high |

ctorse nd Lownship table II. 000000000000000000000000000000000000000000000000000000000000000000000000000000000000000000000000
